# Comparative Analysis of the Complete Chloroplast Genome of Mainland *Aster spathulifolius* and Other *Aster* Species

**DOI:** 10.3390/plants9050568

**Published:** 2020-04-29

**Authors:** Swati Tyagi, Jae-A Jung, Jung Sun Kim, So Youn Won

**Affiliations:** 1 Genomics Division, National Institute of Agricultural Sciences, Rural Development Administration, Jeonju 54874, Korea; swatirajtyagi7@gmail.com (S.T.); jsnkim@korea.kr (J.S.K.); 2 Floriculture Research Division, National Institute of Horticultural and Herbal Science, Rural Development Administration, Wanju 55365, Korea; jabisung@korea.kr

**Keywords:** *Aster*, Asteraceae, chloroplast genome, phylogenetic analysis, codon usage, intron loss

## Abstract

*Aster spathulifolius*, a common ornamental and medicinal plant, is widely distributed in Korea and Japan, and is genetically classified into mainland and island types. Here, we sequenced the whole chloroplast genome of mainland *A. spathulifolius* and compared it with those of the island type and other *Aster* species. The chloroplast genome of mainland *A. spathulifolius* is 152,732 bp with a conserved quadripartite structure, has 37.28% guanine-cytosine (GC) content, and contains 114 non-redundant genes. Comparison of the chloroplast genomes between the two *A. spathulifolius* lines and the other *Aster* species revealed that their sequences, GC contents, gene contents and orders, and exon-intron structure were well conserved; however, differences were observed in their lengths, repeat sequences, and the contraction and expansion of the inverted repeats. The variations were mostly in the single-copy regions and non-coding regions, which, together with the detected simple sequence repeats, could be used for the development of molecular markers to distinguish between these plants. All *Aster* species clustered into a monophyletic group, but the chloroplast genome of mainland *A. spathulifolius* was more similar to the other *Aster* species than to that of the island *A. spathulifolius*. The *accD* and *ndhF* genes were detected to be under positive selection within the *Aster* lineage compared to other related taxa. The complete chloroplast genome of mainland *A. spathulifolius* presented in this study will be helpful for species identification and the analysis of the genetic diversity, evolution, and phylogenetic relationships in the *Aster* genus and the Asteraceae.

## 1. Introduction

*Aster spathulifolius* (Asteraceae) is an economically important perennial herb. It is traditionally used for ornamental purposes, but the aerial part of the plant is also consumed as an edible vegetable or used as herbal medicine to treat several diseases, including asthma, obesity, and diuresis [1,2]. *A. spathulifolius* is native to East Asia, and is specifically distributed along the coastal regions of the Korean Peninsula and Japan [3,4,5]. It thrives in the rocky seashore environment; however, its occurrence in grasslands has also been reported [3,4,5]. 

Understanding genetic diversity, defined as any quantitative metric of genetic variation within a population, is important in many fields of research, including ecology, evolution, genetics, and systematics, as well as for biodiversity conservation and agriculture [6]. In the past, genetic diversity in a wide range of species was estimated by analyzing protein variants known as allozymes [4]. In addition, researchers have scanned the genetic diversity of DNA by utilizing marker information such as amplified fragment length polymorphisms, restriction fragment length polymorphisms, simple sequence repeats (SSRs), and single nucleotide polymorphisms in a single or several genomic loci [4]. The current availability of easy, quick, and cost-efficient technologies such as next-generation sequencing has enabled whole-genome sequencing for the nuclei, mitochondria, and chloroplasts (cp) in many plant species, facilitating the detection of genetic diversity on a genome-wide scale. In particular, cp genomes, due to their highly conserved and stable nature, have been widely used as a comprehensive tool for investigating genetic diversity, evolutionary relationships, molecular phylogenetics, and climatic adaptation, as well as assisting the conservation of rare species and delimiting individuals at the infra-specific level [7,8,9].

The genetic diversity, phylogenetic relationships, and geographic distributions of *A. spathulifolius* populations were previously evaluated using allozymes and inter-SSR (ISSR) markers [4,5]. These populations were genetically differentiated into two distinct groups, mainland and island types, which were consistent with their geographic distributions [5]. The mainland populations showed higher levels of genetic diversity, while island *A. spathulifolius* populations exhibited lower diversity, probably due to their geographic isolation by hundreds of kilometers of ocean and the resulting genetic drift [5]. The complete cp genome for island *A. spathulifolius* was previously sequenced [3,10] and shows relatively high divergence from *Aster altaicus* and *Aster tataricus* [3,10]. Molecular phylogeny methods were also applied to study the wider *Aster* genus, including the analysis of Eurasian *Aster* species using short DNA sequences (ITS, ETS, trnL-F), from which *A. spathulifolius* was excluded [11]. The cp genome of mainland *A. spathulifolius* has not yet been reported, however.

Here, we sequenced the complete cp genome of mainland *A. spathulifolius* and compared it with those of the island *A. spathulifolius* and other available *Aster* species, to explore their molecular identities, genetic divergence, and phylogenetic relationships. This comprehensive and comparative analysis of the cp genomes provides valuable information for conducting a species delimitation, developing molecular markers, and reconstructing the events of *Aster* and *A. spathulifolius* evolution.

## 2. Results and Discussion

### 2.1. Features of the Cp Genome of Mainland A. Spathulifolius

#### 2.1.1. General Structure and Gene Contents

**Figure 1:**
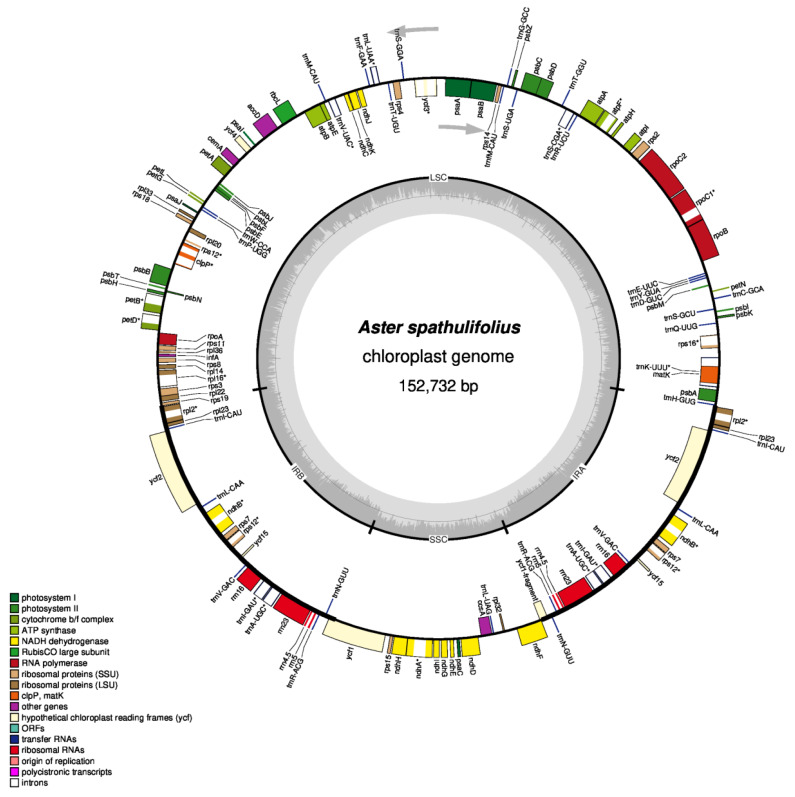
Gene map of the mainland *Aster spathulifolius* chloroplast genome. Thick lines indicate the extent of the inverted repeat regions (IRa and IRb), which separate the genome into the small (SSC) and large (LSC) single-copy regions. Genes drawn inside the circle are transcribed clockwise, while those marked outside the circle are transcribed counterclockwise. Genes belonging to different functional groups are color coded. The dark gray in the inner circle corresponds to the GC content, while the light gray corresponds to the adenine-thymine (AT) content. Genes containing introns are marked with an asterisk. The map was drawn using OGDRAW.

Mainland *A. spathulifolius* had a typical angiosperm cp genome structure and organization (Figure 1). The cp genome was 152,732 bp and was divided into four regions; a pair of inverted repeat (IR) regions (IRa and IRb; 24,956 bp each) separated by two single-copy regions, one large (LSC; 84,552 bp) and one small (SSC; 18,268 bp) (Table 1). The GC contents of the whole cp, LSC, SSC, and IR regions were 37.28%, 35.15%, 31.32%, and 43.06%, respectively (Table 1). The obtained cp genome of mainland *A. spathulifolius* was within the size range (149 to 153 kb) and GC content (37.12 to 37.82%) of the other available cp genomes of the Asteraceae species [12]. Due to the presence of GC-rich ribosomal RNA (rRNA) genes and transfer RNA (tRNA) genes in the IR regions, these genomic sections showed comparatively higher GC contents than the LSC and SSC regions [13]. Indeed, the cp rRNA genes in mainland *A. spathulifolius* had GC contents as high as 55%, and occupied 18.1% of the IR regions. 

The cp genome of mainland *A. spathulifolius* contained 114 non-redundant genes, including four rRNA genes, 30 tRNA genes, and 80 protein-coding genes (Figure 1, Table 2). The LSC region contained 22 tRNA genes and 61 protein-coding genes, whereas the SSC region comprised one tRNA gene and 12 protein-coding genes. Among all these genes, 18 (four rRNA genes, seven tRNA genes, and seven protein-coding genes) were located in the IR regions, and were therefore duplicated. 

A total of 16 genes contained introns, including 14 genes with a single intron and two genes (*clpP* and *ycf3*) with double introns (Table 2). The *clpP* gene encodes a protease involved in translation and post-translational modification processes, while *ycf3* plays an important role in photosynthesis, by regulating the accumulation of the photosystem I complex [14,15]. One gene, *rps12*, encoding *small ribosomal protein 12*, was trans-spliced; its first 5′-end exon was located in the LSC region, while the second exon (3′-end exon) was located in the IRb region and therefore duplicated in IRa. The occurrence of trans-spliced genes, especially *rps12*, is very common among plant cp genomes [8,16]. We also detected seven gene pairs with partially overlapping sequences; *trnK-UUU/matK*, *psbD/psbC*, *atpE/atpB*, *rpoA/rps11*, *rps3/rpl22*, *ndhA/rps12*, and *ndhF/Ψycf1* (Ψ indicates pseudogene). All these pairs had the same gene orientation, with the exception of *ndhF/Ψycf1*, which displayed a reversed orientation.

#### 2.1.2. Codon Usage and Prediction of RNA-Editing Sites

Protein-coding regions (CDS) comprised 51.50% of the mainland *A. spathulifolius* cp genome, and had an average AT content of 61.98% (Table 1). In the 26,218 codons in the protein-coding genes, the AT contents of the first, second, and third bases were 54.33%, 61.89%, and 69.71%, respectively (Table 1). The bias toward a higher AT representation at the third position of the codons has commonly been reported in plant cp genomes, and is believed to distinguish cp DNA from nuclear and mitochondrial DNA [7,8,9]. 

Based on the sequences of the protein-coding genes and tRNA genes, we deduced the amino acid frequency, codon usage, and relative synonymous codon usage (RSCU) in the mainland *A. spathulifolius* cp genome (Table 3). In the cp-encoded proteins, leucine (2815 residues; 10.74%) and cysteine (293 residues; 1.12%) were the most and least prevalent amino acids, respectively. The most and least used codons were AUU (1074 instances; 4.10%; encoding isoleucine) and UGA (15 instances; 0.06%; encoding translational stop), respectively (Table 3). A total of 30 preferred (RSCU > 1) and 32 non-preferred (RSCU < 1) synonymous codons were identified. In general, A- and U-ending codons were the preferred synonymous codons, while the non-preferred codons more commonly ended in C and G, although minor exceptions were observed. No codons were categorized as rare (RSCU < 0.1) in the mainland *A. spathulifolius* cp genome.

**Figure 2:**
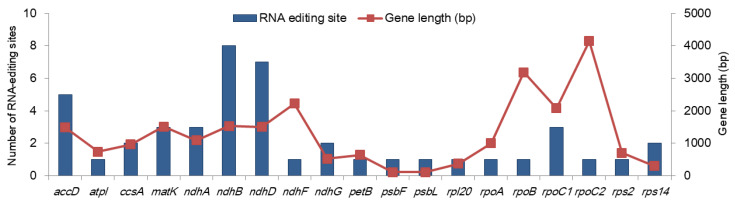
Predicted RNA-editing sites in the protein-coding genes in the mainland *Aster spathulifolius* chloroplast genome. Genes without RNA-editing sites are not plotted.

We predicted 45 RNA-editing sites in 19 genes (Figure 2 and Table S1). All these sites accounted for C-to-U RNA editing, including 36 (80%) editing events in the second position of the codon and 9 (20%) in the first position (Table S1). The highest number of editing sites were detected in *ndhB* (eight events), followed by *ndhD* (seven events) and *accD* (five events). The rest of the genes were predicted to contain 0 to three editing events. No correlation was observed between the gene length and the number of the RNA-editing sites (Figure 2). More than 60% of these RNA-editing events would result in either a serine-to-leucine (17 events; 37.78%) or proline-to-leucine (10 events; 22.22%) conversion (Table S1). No editing events resulted in a stop codon. RNA-editing events occur as a post-transcriptional process to change mRNAs to be translated by generating start codon or removing stop codon and to change the sequences of the proteins (well reviewed in [17]). The RNA folding structure is also changed, which can influence RNA splicing and stability [17]. In plants, the cp genomes have fewer (< 100) RNA-editing sites than the mitochondrial genomes (usually 400–500 events, depending on the size and number of genes) [18]. Our results were consistent with previous studies, in which over 40 RNA-editing sites were reported in the cp genomes [18,19,20]. 

Among the 80 protein-coding genes, only *rps19* employed an alternative start codon (GUG). The genome-encoded ACG for at the start of *ndhD* and *psbL* CDS was predicted to undergo the C-to-U RNA editing to generate the AUG start codon (Table S1). This phenomenon was observed in diverse plant taxa according to the Plant Editosome Database [21]. The protein-coding genes of eukaryotes typically exploit AUG as the start codon; however, in some cases, codons such as AUA, AUC, AUU, and UUG are also used as alternative start codons [8]. This pattern of using alternative start codons is unusual in plants, but has been reported in some species. The *rps19*, *psbC*, and *ycf15* genes use GUG in tobacco (*Nicotiana tabacum*), while *rps19* and *psbL* use ACG as a start codon in rice (*Oryza sativa*) [7,22]. The *ndhD* was also reported to use ACG as a translation initiator in higher plant plastids [23].

### 2.2. Comparative Analysis of the Cp Genomes in the Aster Genus

#### 2.2.1. General Features of the Aster Cp Genomes

The cp genomes of two *A. spathulifolius* populations from different geographic areas (mainland and island) and four other *Aster* species were compared, to elucidate their genomic variation (Table 4). The lengths of these cp genomes varied from 149,473 bp (island *A. spathulifolius*) to 152,992 bp (*A. tataricus*), with mainland *A. spathulifolius* having the third largest cp genome. The island *A. spathulifolius* possessed the smallest cp genome among all 211 sequenced cp or plastid genomes from the Asteraceae [12]. The total number of non-redundant genes was conserved among the *Aster* species. Exceptionally, the *atpB* gene of island *A. spathulifolius* had a 4-bp duplication (AATT) after the first three codons, which prevented the proper annotation of *atpB* in the original database. Given that the *atpB* gene is essential for photosynthesis and the deletion of one duplicate resulted in the *atpB* CDS identical to those in other *Aster* species, *atpB* was not excluded from the cp gene set in this study and was subjected for all comparative analyses. 

The *Aster* cp genomes commonly possessed 18 intron-containing genes (six tRNA genes and 12 protein-coding genes) (Table 5). However, the *Aster* species showed variation in the intron size, except for *rpl2*, *rps12*, and *trnV-UAC*. The *rps16* gene showed the largest size difference of 127 bp, which was followed by *petB* of 95 bp and *petD* of 85 bp. The introns in cp genomes ranged from 423 bp to 2539 bp in length. The longest intron (2502–2542 bp) was observed in *trnK-UUU*, and accommodated *matK* within it. 

#### 2.2.2. Cp Genome Sequence Conservation and Divergence

**Figure 3:**
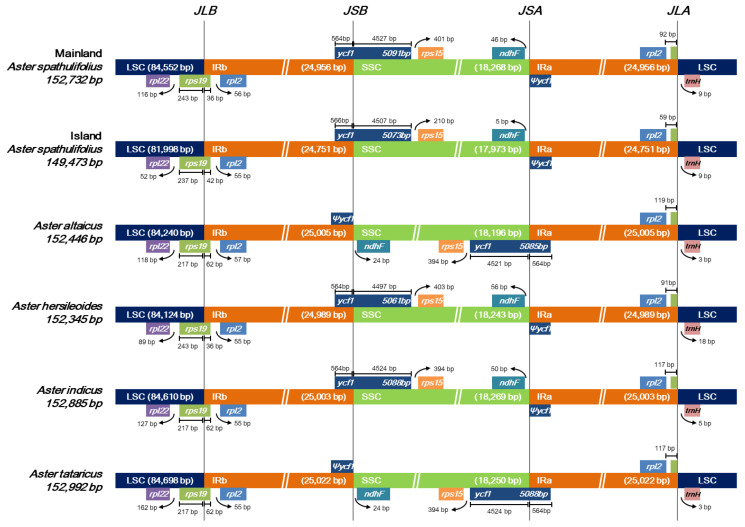
Comparison of large single-copy regions (LSC), inverted repeat (IR), and small single-copy regions (SSC) junction positions among six *Aster* chloroplast genomes. Ψ indicates a pseudogene. Genes above the longer box are transcribed in rightward direction and genes below the longer box are transcribed in leftward direction.

A pairwise alignment of the cp genomes revealed a large-scale inversion of the entire SSC region in *A. altaicus* and *A. tataricus*, as shown in the altered gene positions at the two SSC/IR junctions (Figure 3). This is a common event in the cp genome, and is consistent with the hypothesis that cp DNA can exist in two orientations at the SSC region [24]. 

**Figure 4:**
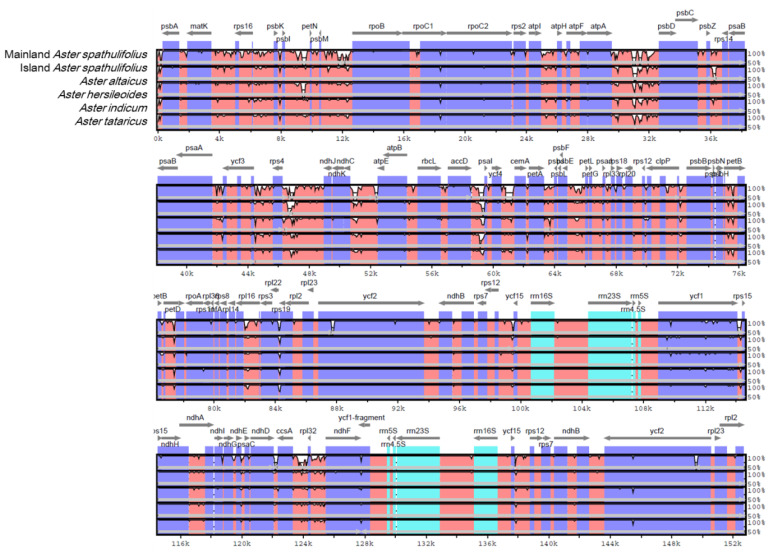
Comparison of the chloroplast genome of mainland *Aster spathulifolius* with those of other related species. SSC region in *A. altaicus* and *A. tataricus* was reoriented for comparison. Gray arrows above the alignment indicate the orientation of genes. Dark blue bars represent protein-coding genes, pale blue bars represent rRNA genes, and red bars represent conserved non-coding sequences. A cut-off of a 70% identity was used for the plots. The y-scale axis represents the percentage identity (50–100%). mVISTA was used to perform the comparison.

A comparison of the cp genomes was used to explore their DNA sequence conservation and synteny (the gene order across the genomes). For this, the SSC sequences for *A. altaicus* and *A. tataricus* were reoriented. A BLAST-based CGView Comparison Tool (CCT) analysis showed that the mainland *A. spathulifolius* cp genome shared high levels of sequence similarity (> 90%) with all the *Aster* species analyzed (Figure S1). The mVISTA analysis indicated that the IR regions were more conserved than the single-copy regions (Figure 4). Genetic variability was more prevalent in the intergenic or non-coding regions than in the coding regions. The highly divergent regions included *rps16_rpoB*, *atpI_atpF*, *atpA_psbD*, *psbZ_rps14*, *ycf3_ndhJ*, *ndhK_atpE*, *accD_psaI*, *ycf4_cemA*, *petA_petJ, psbE_rpl20*, *psbH_petB*, *petB_petD*, *rpl16_rps19*, *ycf1_rps15*, *ndhI_ndhD*, and *ccsA_ndhF*. In comparison to mainland *A. spathulifolius*, island *A. spathulifolius* contained more regions showing divergence than the other *Aster* species did; however, our intra-genus comparison revealed less divergence than was reported in a previous analysis, comparing the island *A. spathulifolius* cp genome with the more distant members of the Asteraceae family [3].

We compared the nucleotide diversity in the complete *Aster* cp genomes, as well as the LSC, SSC, and IR regions (Table S2). Although the sequence alignment revealed a high sequence similarity, 2289 variable sites (1.55%) were detected in the complete cp genomes, 224 (9.78%) of which were parsimony-informative sites. The nucleotide diversity in the complete cp genome was 0.00563. Consistent with the above sequence alignment (Figure 4), the LSC and SSC regions (0.00776 and 0.00884, respectively) had a 10-fold greater nucleotide diversity than the IR regions (0.00077) (Table S2). In accordance with this, the single-copy regions exhibited a higher number of mutations and InDels than the IR regions.

#### 2.2.3. Gene-By-Gene Sequence Divergence

**Figure 5:**
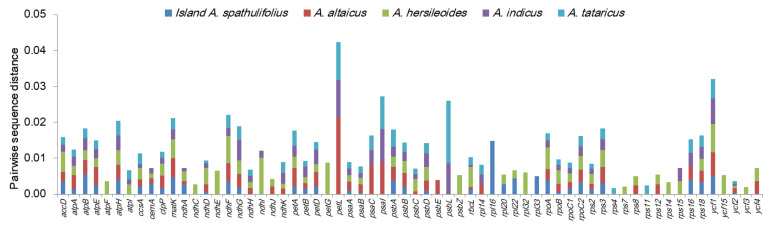
Pairwise sequence distances of the protein-coding genes in the chloroplast genomes of mainland *Aster spathulifolius* and related species. The mainland *A. spathulifolius* chloroplast genome was compared with those of the island *A. spathulifolius*, *A. altaicus*, *A. hersileoides*, *A. indicus*, and *A. tataricus*. Of the 80 protein-coding genes analyzed, the 64 showing noticeable divergences are displayed here. Genes with zero divergence were omitted.

The pairwise sequence divergence between the mainland *A. spathulifolius* and other *Aster* plants was calculated for the 80 common protein-coding genes (Figure 5). Many genes were well conserved, while others showed clear sequence differences. The ten most divergent genes were *petL*, *ycf1*, *psaI*, *psbL*, *ndhF*, *matK*, *atpH*, *ndhG*, *rps3*, and *atpB* (in decreasing order). With the exception of *ycf1*, which was located in both IR and SSC, all the divergent genes were located in the LSC and SSC regions, and displayed a trend toward more rapid evolution [7]. 

**Figure 6:**
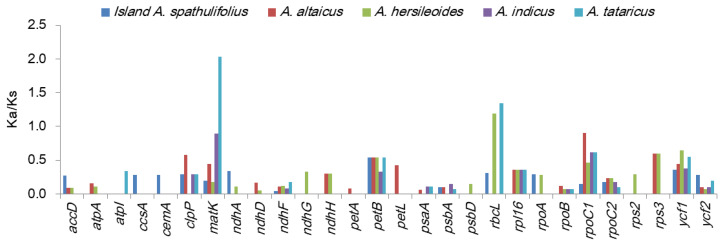
Ka/Ks values of protein-coding genes in the *Aster* species. Mainland *A. spathulifolius* was compared with island *A. spathulifolius*, *A. altaicus*, *A. hersileoides*, *A. indicus*, and *A. tataricus*. Genes with zero Ka/Ks value in all comparisons were omitted.

The selection pressure was also examined by calculating the nonsynonymous (Ka) and synonymous (Ks) substitution rates and the Ka/Ks ratio between the mainland *A. spathulifolius* and the other *Aster* plants (Figure 6, Table S3). In all comparisons, the Ka values ranged from 0 to 0.0150 and the Ks values ranged from 0 to 0.0441 (Table S3). Overall, *atpA*, *atpB*, *atpH*, *ccsA*, *cemA*, *ndhD*, *psaA*, *psbD*, *rpoC1* and *rps3* had high levels of synonymous substitutions, while *atpH*, *matK*, *petB*, *petL*, *rbcL*, *rpl16*, *rps12*, *rps16*, *rps3*, and *ycf1* had high levels of nonsynonymous changes. Several genes such as *infA*, *ndhB*, *petN*, *psaJ*, *psbF*, *psbH*, *psbI*, *psbJ*, *psbK*, *psbM*, *psbN*, *psbT*, *rpl2*, *rpl23*, *rpl36*, *rps7*, *rps18*, *rps19*, and *ycf15* were not observed to have any synonymous or nonsynonymous changes. Most of the Ka/Ks ratios were less than 1, indicating that the cp genes were under purifying selection (Figure 6, Table S3). Only *matK* and *rbcL* in *A. tataricus* and *rbcL* in *A. hersileoides* were under positive selection, as shown by their Ka/Ks values greater than 1. From a less stringent perspective, five genes (*clpP*, *petB*, *rpoC1*, *rps3*, and *ycf1*) in the several comparison pairs had Ka/Ks values ranging from 0.5 to 1, indicating a mild positive selection. Between the two *A. spathulifolius* populations, all of the Ka/Ks ratios were less than 0.5, except for *petB* (0.5426). The genes under positive selection were generally located in the LSC region, except for *ycf1* located in the IR and SSC region. Among the genes under positive selection, two genes (*matK* and *ycf1*) were previously reported to experience this type of selection in other taxonomic groups, with *matK* being reported to be under positive selection in more than 30 groups [25,26].

#### 2.2.4. Contraction and Expansion of the IR Region

The expansion and contraction of the IR region are major evolutionary events that influence the lengths of cp genomes [27]. To examine these processes in the *Aster* species, we dissected the LSC/IRb, IRb/SSC, SSC/IRa, and IRa/LSC junctions for all six *Aster* sequences (Figure 3). In all cases, *rps19* was shared by the LSC and IRb regions. The length of *rps19* was quite conserved at 279 bp; however, a shift of this gene towards the LSC region was observed in certain species. In *A. altaicus, A. indicus*, and *A. tataricus*, 62 bp of *rps19* was shared by the IR region, whereas in both the mainland and island *A. spathulifolius* lines, this was reduced to 36 and 42 bp, respectively, and thus the gene tended to move towards the LSC region. Since *A. altaicus* and *A. tataricus* exhibited an inversion of the SSC region, the genes at the IRb/SSC and SSC/IRa junctions were affected in these species. The IRb/SSC junction was shared by the functional *ycf1* gene in mainland *A. spathulifolius*, island *A. spathulifolius*, *A. hersileoides*, and *A. indicus*, compared with the shorter *ycf1* pseudogene detected in *A. altaicus* and *A. tataricus*. At the SSC/IRa junction, *ndhF* and *Ψycf1* were identified in mainland *A. spathulifolius*, island *A. spathulifolius*, *A. hersileoides*, and *A. indicus*, whereas *A. altaicus* and *A. tataricus* possessed the functional copy of *ycf1*. Finally, *rpl2* and *trnH-GUG* were commonly located at the IRa/LSC boundary, with variable gap lengths observed in the six *Aster* cp genomes. 

Pseudogenes are nonfunctional copies of genes generated through loss-of-function mutations, such as frameshift mutations, premature stop codons, or distortions of regulatory sequences [28]. In some cases, pseudogenes arise through the inadequate duplication of parental genes [29]. Similar mutations were observed in the *Aster* cp genomes, in which three pseudogenes (*rps19, ycf1*, and *ycf68*) were identified. The *rps19* and *ycf1* genes shared the LSC/IRb and SSC/IRb boundaries, respectively (Figure 3), and the duplication of the IR region resulted in the formation of partial copies of these genes (*Ψrps19, Ψycf1*). In the mainland and island *A. spathulifolius*, *A. hersileoides*, and *A. indicus* cp genomes, the 5’ region of the *ycf1* gene, including the start codon, was located in the IRb region, while the remaining 3’ region, covering around 90% of the complete *ycf1*, extended into SSC, causing a duplication of the 5′ portion of *ycf1* in IRa. Consequently, a ~-567bp *Ψycf1* sequence was observed in IRb, instead of the ~5,094-bp functional copy. The pseudogenization of *ycf68* occurred in the six *Aster* cp genomes, due to the presence of several premature stop codons in its coding sequence, which was consistent with a pattern of pseudogene formation reported in other studies [3,10]. Pseudogenes usually evolve neutrally; however, some studies revealed that they can be transformed into another gene with a different function, after experiencing strong purifying selection pressure [30]. 

#### 2.2.5. Repeat Sequence Analysis

**Figure 7:**
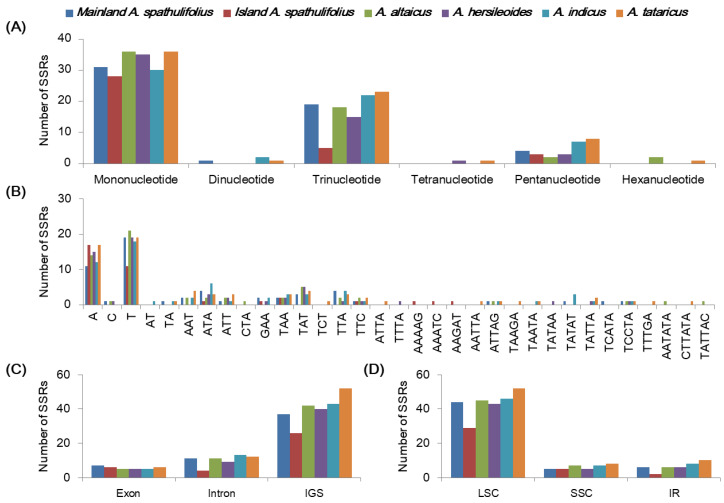
Analyses of the simple sequence repeats (SSRs) in the *Aster* chloroplast genomes. (**A**) The frequency of simple sequence repeats (SSRs) by unit length in six *Aster* chloroplast genomes. (**B**) The frequency of SSRs per sequence type. (**C**) Frequency of SSRs in exon, intron, and intergenic spacer (IGS). (**D**) Frequency of SSRs in large single copy (LSC), small single copy (SSC), and inverted repeat (IR) regions.

Microsatellites, commonly known as SSRs, are short (1–6 nucleotides) tandem unit sequences. They exhibit polymorphisms in their copy numbers and therefore lengths due to replication slippage, and have frequently been used as molecular markers in plant evolution studies [9]. Our SSR analysis detected 55, 36, 58, 54, 61, and 70 SSRs in the mainland *A. spathulifolius*, island *A. spathulifolius*, *A. altaicus, A. hersileoides, A. indicus*, and *A. tataricus* cp genomes, respectively (Table S4). The cp SSRs showed conservation and variation among the *Aster* species. In terms of the unit size, a mononucleotide repetition was the most frequent SSR in the analyzed species, followed by tri- and pentanucleotide SSRs (Figure 7A). In mainland *A. spathulifolius*, 56.4% of SSRs were formed of mononucleotide units, varying from 10 to 17 nucleotide iterations and with adenine or thymine representing the most frequent base (Figure 7B). The SSRs were largely distributed in the intergenic spacers (IGS) (67.3%–74.3% of total SSRs) and LSC regions (74.3%–80.6%) (Figure 7C,D). Our results were consistent with the hypothesis that cpSSRs are generally composed of short polyadenine or polythymine repeats and rarely contain tandem guanine or cytosine repeats [7]; thereby contributing to the AT richness of the cp genome. Variation in the number and type of cpSSRs is common between species [10,31] and could be used to detect polymorphisms between species.

**Figure 8:**
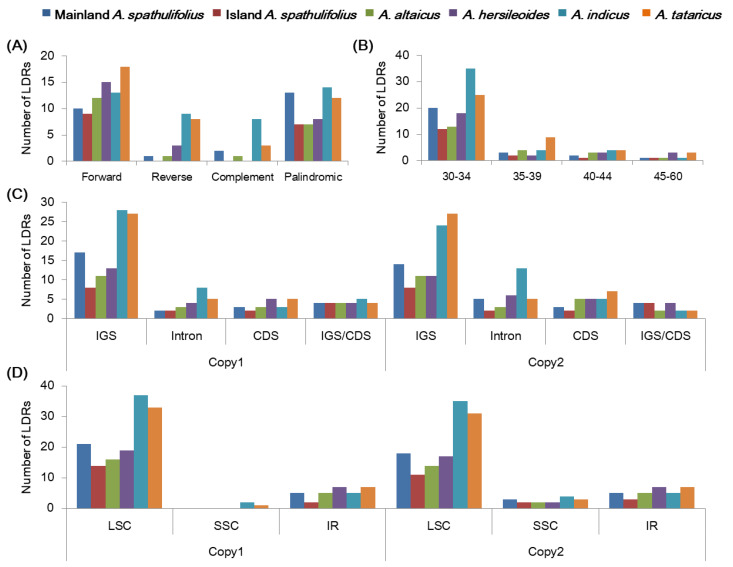
Analyses of the long-dispersed repeats (LDRs) in the *Aster* chloroplast genomes. (**A**) Frequency of four LDR types. (**B**) Frequency of LDRs by length. (**C**) Frequency of LDRs in intergenic spacer (IGS), coding region (CDS), and intron. IGS/CDS represents repeats shared in IGS and CDS. (**D**) Frequency of LDRs in large single copy (LSC), small single copy (SSC), and inverted repeat (IR) regions. The frequency in (**C**,**D**) was determined for the first copy and second copy separately.

Longer dispersed repeats (LDRs) were detected and classified into four categories, forward, reverse, complement, and palindromic repeats, with a motif length of 30–60 bp in the *Aster* cp genomes using the REPuter program (Table S5) [32]. The *Aster* cp genomes showed differences in the number of total LDRs, the frequencies of each class, their lengths, and distribution. In total, 26, 16, 21, 26, 44, and 41 LDRs were detected in the mainland *A. spathulifolius*, island *A. spathulifolius*, *A. altaicus, A. hersileoides, A. indicus*, and *A. tataricus* cp genomes, respectively. The cp genomes contained more forward and palindromic repeats than reverse and complement repeats (Figure 8A). In the mainland *A. spathulifolius* cp genome, we identified 10 forward, 1 reverse, 2 complement, and 22 palindromic repeats. In addition, repeats with 30–34 bp in length were the most common in the *Aster* species (Figure 8B). Most of these LDRs were present in the IGS, but several were observed within the CDS regions and introns (Figure 8C). The *ndhF*, *psaB*, *psbN*, *ycf2*, *trnS-UGA*, *trnS-GCU* genes contained LDRs. The LDRs were also detected more in LSC than in SSC and IR regions (Figure 8D). The overall distribution of LDRs was similar in both copies (Figure 8C,D). Our results are supported by previous findings that the LDRs were more prevalent in the non-coding regions than the coding regions [10,33,34]. 

#### 2.2.6. Phylogenetic Studies 

**Figure 9:**
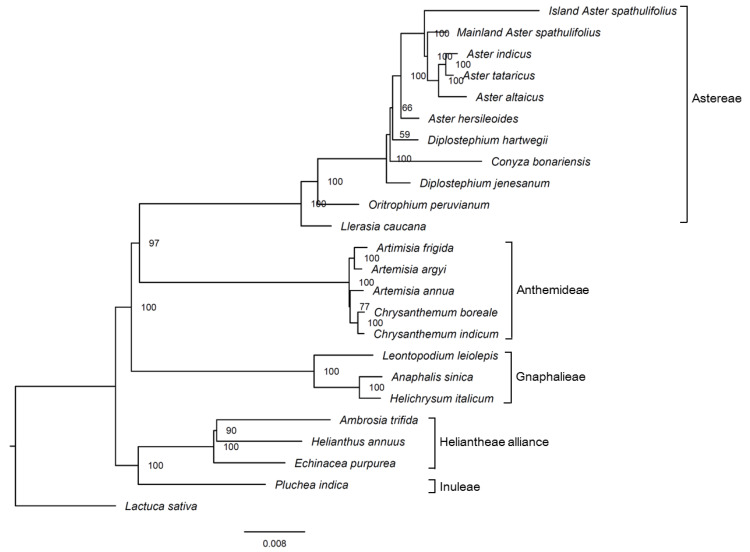
Phylogenetic tree of the complete chloroplast genomes of various Asteraceae members, generated using the maximum likelihood method. Numbers at nodes indicate bootstrap values (1000 repeats). The scale bar represents the expected number of nucleotide substitutions per site. *Lactuca sativa* was used as the outgroup.

The maximum likelihood (ML) phylogenetic tree was constructed using the whole cp sequences from 23 species in the subfamily Asteroideae (Asteraceae), using lettuce (*Lactuca sativa*; Cichorioideae, Asteraceae) as an outgroup (Figure 9, Table S6). The Asteroideae species formed a monophyletic group and were separated into several clusters, each of which represented the tribes Astereae, Anthemideae, Gnaphalieae, Inuleae, and the Heliantheae alliance. The mainland *A. spathulifolius* sequence was clustered together with the other *Aster* species, including island *A. spathulifolius*, supported by a 100% bootstrap value. In the previous analysis with cp genomes, island *A. spathulifolius* cp genome was clustered with non-*Aster* species, *Conyza bonariensis* and *Lagenophora cuchumatanica*, while *A. altaicus* and *A. tataricus* were clustered into a monophyletic group [10].

A pairwise sequence divergence analysis of the complete cp genomes revealed that mainland *A. spathulifolius* was more distant from island *A. spathulifolius* (0.01214) than it was from the other *Aster* species, including *A. tataricus* (0.00287), *A. indicus* (0.00296), *A. altaicus* (0.00388), and *A. hersileoides* (0.00434) (Table S7). This is consistent with the result from phylogenetic analysis. To correctly delimit this species, we need to examine whether the geographical isolation of *A. spathulifolius* resulted in its reproductive isolation. In addition, the genetic distance and molecular phylogeny should be further addressed, using more markers in the nucleus and larger sample sizes. 

In addition to cp genome, the nuclear ribosomal internal transcribed spacer regions (nrITS) have been extensively used as molecular markers for species identification and phylogenetic analysis. We revealed the nrITS sequence using the same procedure for cp genome and conducted phylogenetic analysis with the available nrITS sequences of *A. spathulifolius* populations and other *Aster* species (Figure S2). It was found that seven *A. spathulifolius* lines, including the mainland line, formed a monophyletic group and classified into two clades. Our mainland line was clustered with four *A. spathulifolius* lines, three of which were collected from three different locales in mainland [35] and one from unknown region. Another clade included two *A. spathulifolius* lines and *Aster oharai*. Two nrITS sequences in another clade were deposited in NCBI by that which characterized the cp genome of island line addressed here. Interestingly, *A. oharai* is distributed only in Dokdo island, the collection site of island *A. spathulifolius*, and its neighboring Ulleungdo island, and resembles *A. spathulifolius* [35]. The taxonomical position of *A. oharai* is not clear: it is regarded as the synonym of *A. spathulifolius* [36] or as a variety of *A. spathulifolius* [37]. Although the taxonomical status of *A. spathulifolius* and *A. oharai* needs to be further examined, our comparative and phylogenetic analyses suggest that the island line is more likely to be classified into *A. oharai* rather than *A. spathulifolius*. 

#### 2.2.7. Positive Selected Analysis

**Figure 10:**
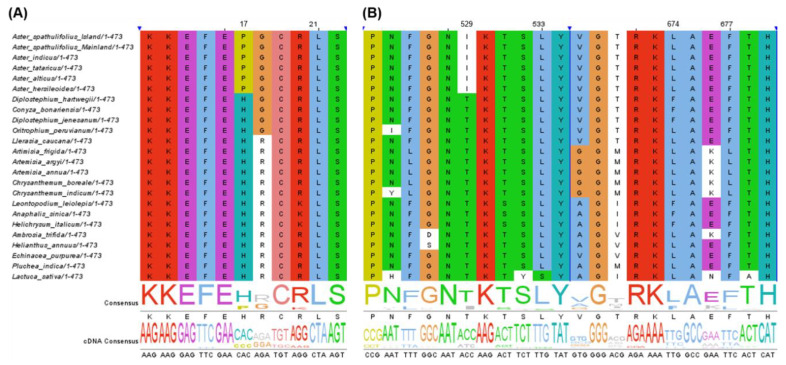
The partial alignment of positively selected genes in the *Aster* species. Two genes were determined by branch-site model and Bayesian Empirical Bayes test. (**A**) The partial alignment of amino acid sequences of *accD*. (**B**) The partial alignment of amino acid sequences of *ndhF*.

The protein-coding genes under positive selection within the *Aster* species compared to non-*Aster* species were identified based an optimized branch-site model [38] and Bayesian Empirical Bayes (BEB) methods [39]. No significant positive selection (*p*-value > 0.05) was observed in any genes (Table S8). However, two genes (*accD* and *ndhF*) showed significant posterior probabilities indicating positively selected sites according to the BEB test (Table S8). The *Aster* lineage specifically possessed proline at the amino acid position 17 of *accD* and isoleucine at 529 and leucine at 674 of *ndhF* (Figure 10). The positive selection on *accD* and *ndhF* was detected within the other plant lineages, including *Cardamine* [40] and Cucurbitaceae [41]. The *accD* gene encodes the β-carboxylase subunit of acetyl-CoA carboxylase and is responsible for catalysis of the first and rate-limiting step of lipid biosynthesis [42]. The *accD* gene was previously reported to influence leaf development and seed yield [43,44]. The *ndhF* gene encodes one subunit of NAD(P)H dehydrogenase (NDH) complex, and is responsible for electron transport chain to generate ATP in the photosynthesis process [45,46]. The *ndhF* gene was characterized to be under positive selection in the Australian lineage of *Citrus* and to contribute the adaptation to the hot and dry climate [47]. Although two genes showed significant mark of positive selection, the adaptive evolution and divergence of the *Aster* species in the specific ecological environment needs to be further addressed by molecular, physiological and ecological studies.

## 3. Materials and Methods 

### 3.1. Ethics Statement

The plant sample used in this study is neither endangered nor protected, and was collected from an area that was not privately owned or protected in any way. No specific permits were required to conduct this study. 

### 3.2. Sample Collection and Sequencing

*A. spathulifolius* was collected from Yeoju-si, Gyeonggi-do, Republic of Korea (37°14′18′′ N, 127°41′10′′ E) and deposited in the National Agrobiodiversity Center, Rural Development Administration, under the accession number IT317814. Since the obtained plant was collected from the Korean peninsula, it is referred to as “mainland *A. spathulifolius*” throughout this manuscript, to distinguish it from the previously reported cp genome of *A. spathulifolius* native to Dokdo island, Republic of Korea [3]. 

Genomic DNA was extracted from fresh leaves as previously described [48]. The quantity and integrity of the DNA were checked using a spectrophotometer (NanoDrop 2000; Thermo Fisher Scientific, Waltham, MA, USA), gel electrophoresis on a 0.8% agarose gel, and a PicoGreen assay (Thermo Fisher Scientific, Waltham, MA USA). Using high-quality DNA, a 350-bp paired-end library was prepared using a TruSeq DNA PCR-Free kit (Illumina, San Diego, CA USA), after which it was sequenced at Macrogen (Republic of Korea) using an Illumina HiSeq4000 (Illumina, San Diego, CA, USA) with a 101-bp read length, following the manufacturer’s protocol.

### 3.3. Cp Genome Assembly, Annotation, and Sequence Analysis 

The complete cp genome was determined using the dnaLCW (de novo assembly of low coverage whole-genome shotgun sequences) procedure [33]. Briefly, raw genomic reads were pre-processed using a Trimmomatic program [49], assembled using clc_assembler in CLC Genomics Workbench v6.0 (CLC Bio, Denmark) and gap-filled using GapCloser [50]. Cp-encoding contigs were BLASTn-searched against the cp genome of another Asteraceae species, *Chrysanthemum boreale* (NCBI ID MG913594) [51], and circularized by comparing the end sequences. 

The cp sequences were annotated using Dual Organellar GenoMe Annotator (DOGMA), cpGAVAS v2.0, GeSeq and BLAST searches, and the start and stop codons were further checked and manually corrected if needed [52,53]. The tRNAscan-SE 2.0 (URL: http://lowelab.ucsc.edu/tRNAscan-SE/) was used with default settings, to predict and verify the tRNA structure [54]. OGDRAW v1.2 was used to generate a circular genome map with structural features [55]. The obtained cp genome was deposited in the National Center for Biotechnology Information (NCBI), under the accession number MN539621.

The codon usage bias was calculated using MEGA X [56]. For the protein-coding genes, the possible RNA-editing sites were predicted using the Predictive RNA Editor for Plant cp genes (PREP-Cp), with a cut-off score of 0.8 [57,58].

### 3.4. Comparative Genomic Analysis and Evaluation of highly Divergent Regions 

The following complete cp genomes of five *Aster* species were downloaded from the NCBI database: island *A. spathulifolius* (KF279514), *A. altaicus* (NC_034996.1), *A. hersileoides* (MK290823), *A. indicus* (NC_040126.1), and *A. tataricus* (MH669275). After comparing gene sets between species, the genes that did not exist in the original annotation were searched by BLAST analysis. 

The nucleotide diversities, sequence polymorphisms, and genome divergence among six *Aster* genomes were analyzed using DnaSP v6 [59]. The Ka and Ks substitutions, as well as the Ka/Ks ratio, were analyzed for each protein-coding gene pair between the mainland *A. spathulifolius* and other *Aster* sequences using DnaSP v6. Furthermore, the mainland *A. spathulifolius* cp genome was compared with the other cp genomes using the CCT [60]. Briefly, the cp genomes were compared using BLAST, then aligned and plotted as a circular map. GC distributions were measured on the basis of the GC skew, using the following equation: GC skew = (G − C)/(G + C). The pairwise sequence divergence of complete cp genome and protein-coding genes was calculated using MEGA X [56]. The mVISTA program was used in the Shuffle-LAGAN mode, to compare these cp genomes with the annotated mainland *A. spathulifolius* sequence [61].

### 3.5. Characterization of Repeat Sequences

SSRs were discovered using the online web tool MISA (http://pgrc.ipk-gatersleben.de/misa/) with the following parameters: ten for mononucleotide motifs, eight for di-nucleotide, four for tri- and tetra-nucleotide, and three for penta- and hexa-nucleotide motifs [62]. The REPuter program was used to identify the four different types of repeats (forward, reverse, complement, and palindromic repeats), using a minimum repeat size of 30 bp, a sequence identity > 90%, and Hamming distance of three [32]. To avoid redundancy, one copy of IR regions (IRa) was removed prior to LDR analysis.

### 3.6. Phylogenetic Analysis 

The complete cp genomes of 23 species from the Asteraceae family were downloaded from the NCBI database and compared with the cp genome of the mainland *A. spathulifolius* plant, using BLASTn to check the orientation of the SSC sequences. For species with reversed SSC regions, the SSC sequences were reoriented. All the cp genomes were aligned using the Clustal Omega program [63] and subjected to a ML analysis using the IQ-TREE web server, with the model of TVM + F + I + G4 and 1000 bootstrap replicates. 

### 3.7. Positive Selected Analysis

To identify the selection pressure on cp genes, an optimized branch-site model [38] and BEB methods [39] were used. The CDS sequences of all taxa were aligned using MUSCLE, with the option of codon alignment in MEGA X program [56] and exported into phylip format without gap sequences. The selection pressure was calculated with codeml in PAML v4.8 package [64]. The branch-site model was implemented by defining the *Aster* genus as foreground branch. The selection pressure was calculated into the Ka/Ks ratio named as ω. The value ω > 1, ω = 1, ω < 1 represents positive selection, neutral evolution, and purifying selection, respectively [65]. The log-likelihood values were calculated and tested [66]. The posterior probabilities of amino acid were calculated by the BEB method, to identify specific position under positive selection [39]. The gene with a significant test p-value < 0.05 or with positively selected sites was considered to be under positive selection. The amino acid sequences of positively selected genes were visualized with Jalview v.2.11 [67].

## Figures and Tables

**Table 1:** Base composition and length of regions of the mainland *Aster spathulifolius* chloroplast genome.

Category	T/U (%)	C (%)	A (%)	G (%)	Length (bp)
Genome	31.53	18.37	31.19	18.91	152,732
LSC	32.61	17.27	32.23	17.88	84,552
SSC	34.87	14.82	33.81	16.50	18,268
IRa	28.62	20.79	28.32	22.28	24,956
IRb	28.32	22.28	28.62	20.79	24,956
tRNA genes	25.00	23.56	22.09	29.35	2784
rRNA genes	18.79	23.63	26.04	31.54	9048
Protein-coding genes	31.45	17.72	30.53	20.31	78,654
1st position	23.82	18.87	30.51	26.80	26,218
2nd position	32.58	20.31	29.30	17.81	26,218
3rd position	37.93	13.98	31.78	16.31	26,218

**Table 2:** List of genes in the mainland *Aster spathulifolius* chloroplast genome.

Category	Group of Genes	Name of Genes
Self-replication	Large subunit of ribosomal proteins	rpl2 * (2x), 14, 16 *, 20, 22, 23(2x), *32, 33, 36*
	Small subunit of ribosomal proteins	*rps2, 3, 4, 7*(2x), 8, 11, 12 ** (2x), 14, 15, 16 *, 18, 19
	DNA dependent RNA polymerase	rpoA, B, C1 *, C2
	rRNA genes	*rrn16S*(2x) *, rrn23S*(2x)*, rrn4.5S*(2x)*, rrn5S*(2x)
	tRNA genes	trnA-UGC * (2x), trnC-GCA, trnD-GUC, trnE-UUC, trnF-GAA, trnfM-CAU, trnG-GCC, trnG-UCC *, trnH-GUG, trnI-CAU(2x), trnI-GAU * (2x), trnK-UUU *, trnL-CAA(2x), trnL-UAA *, trnL-UAG, trnM-CAU, trnN-GUU(2x)*, trnP-UGG, trnQ-UUG, trnR-ACG*(2x)*, trnR-UCU, trnS-GCU, trnS-GGA, trnS-UGA, trnT-GGU, trnT-UGU, trnV-GAC*(2x), trnV-UAC *, trnW-CCA, trnY-GUA
Photosynthesis	Photosystem I	*psaA, B, C, I, J*
	Photosystem II	*PsbA, B, C, D, E, F, H, I, J, K, L, M, N, T, Z*
	NADH dehydrogenase	ndhA *, B * (2x), *C, D, E, F, G, H, I, J, K*
	Cytochrome b6/f complex	petA, B * , D * *, G, L, N*
	ATP synthase	atpA, B, E, F *, H, I
	Rubisco	*rbcL*
Other genes	Translational initiation factor	*infA*
	Maturase	*matK*
	Protease	clpP *
	Envelop membrane protein	*cemA*
	Subunit acetyl-CoA carboxylase	*accD*
	C-type cytochrome synthesis gene	*ccsA*
Unknown	Conserved open reading frame	*ycf1*, *2*(2x), 3 *, 4, 15(2x)

* Genes containing introns; ** Trans-spliced genes; (2X) indicates copy numbers.

**Table 3:** Relative synonymous codon usage (RSCU), following the codon frequency in the protein-coding sequences of the mainland *Aster spathulifolius* chloroplast genome.

Amino Acid	Codon	No	RSCU	tRNA	Amino Acid	Codon	No	RSCU	tRNA
Phe	UUU	983	1.31		Thr	ACU	529	1.61	
Phe	UUC	516	0.69	*trnF-GAA*	Thr	ACC	238	0.73	*trnT-GGU*
Leu	UUA	865	1.84	*trnL-UAA*	Thr	ACA	406	1.24	*trnT-UGU*
Leu	UUG	588	1.25	*trnL-CAA*	Thr	ACG	138	0.42	
Leu	CUU	607	1.29		Ala	GCU	624	1.74	
Leu	CUC	194	0.41		Ala	GCC	236	0.66	
Leu	CUA	379	0.81	*trnL-UAG*	Ala	GCA	408	1.14	*trnA-UGC*
Leu	CUG	182	0.39		Ala	GCG	166	0.46	
Ile	AUU	1074	1.47		Tyr	UAU	802	1.64	
Ile	AUC	427	0.58	*trnI-GAU*	Tyr	UAC	177	0.36	*trnY-GUA*
Ile	AUA	689	0.94		STOP	UAA	51	1.76	
Met	AUG	635	1.00	*trn(f)M-CAU*	STOP	UAG	21	0.72	
Val	GUU	511	1.46		STOP	UGA	15	0.52	
Val	GUC	178	0.51	*trnV-GAC*	His	CAU	457	1.50	
Val	GUA	524	1.49	*trnV-UAC*	His	CAC	153	0.50	*trnH-GUG*
Val	GUG	191	0.54		Gln	CAA	727	1.53	*trnQ-UUG*
Ser	UCU	590	1.77		Gln	CAG	221	0.47	
Ser	UCC	311	0.93	*trnS-GGA*	Asn	AAU	973	1.53	
Ser	UCA	403	1.21	*trnS-UGA*	Asn	AAC	298	0.47	*trnN-GUU*
Ser	UCG	169	0.51		Lys	AAA	1034	1.48	*trnK-UUU*
Ser	AGU	401	1.21		Lys	AAG	366	0.52	
Ser	AGC	122	0.37	*trnS-GCU*	Asp	GAU	851	1.61	
Arg	AGA	495	1.87	*trnR-UCU*	Asp	GAC	209	0.39	*trnD-GUC*
Arg	AGG	174	0.66		Glu	GAA	990	1.48	*trnE-UUC*
Arg	CGU	353	1.33	*trnR-ACG*	Glu	GAG	352	0.52	
Arg	CGC	109	0.41		Cys	UGU	207	1.41	
Arg	CGA	346	1.31		Cys	UGC	86	0.59	*trnC-GCA*
Arg	CGG	113	0.43		Trp	UGG	462	1.00	*trnW-CCA*
Pro	CCU	409	1.48		Gly	GGU	574	1.29	
Pro	CCC	211	0.76		Gly	GGC	199	0.45	*trnG-GCC*
Pro	CCA	316	1.14	*trnP-UGG*	Gly	GGA	684	1.53	*trnG-UCC*
Pro	CCG	170	0.61		Gly	GGG	329	0.74	

**Table 4:** Summary of the complete chloroplast genomes of the six *Aster* species used in this study.

Attributes	Mainland *A. spathulifolius*	Island *A. spathulifolius*	*A. altaicus*	*A. hersileoides*	*A. indicus*	*A. tataricus*
Size (bp)	152,732	149,473	152,446	152,345	152,885	152,992
GC content (%)	37.2	37.7	37.3	37.3	37.0	37.3
LSC size (bp)	84,552	81,998	84,240	84,124	84,610	84,698
SSC size (bp)	18,268	17,973	18,196	18,243	18,269	18,250
IR size (bp)	24,956	24,751	25,005	24,989	25,003	25,022
No. of genes	114	114	114	114	114	114
Protein-coding genes	80	80	80	80	80	80
tRNA genes	30	30	30	30	30	30
rRNA genes	4	4	4	4	4	4
Genes duplicated	18	18	18	18	18	18
Genes with intron	17	17	17	17	17	17
Pseudogene	3	3	2	2	2	2

**Table 5:** Comparison of intron lengths in the mainland *Aster spathulifolius* chloroplast genome with those of other related species.

Genes		Location	Mainland *A. spathulifolius*	Island *A. spathulifolius*	*Aster altaicus*	*Aster hersileoides*	*Aster indicus*	*Aster tataricus*
*atpF*		LSC	715	699	709	709	709	718
*clpP*	Intron1 Intron2	LSC	812	800	812	811	814	812
			616	623	614	615	615	614
*ndhA*		SSC	1064	1105	1046	1077	1064	1055
*ndhB*		IR	676	670	674	674	674	674
*petB*		LSC	840	745	798	796	823	830
*petD*		LSC	767	724	760	785	809	745
*rpl16*		LSC	1058	1017	1087	1053	1098	1144
*rpl2*		IR	671	671	671	671	671	671
*rpoC1*		LSC	742	721	743	742	742	743
*rps12*			535	535	535	535	535	535
*rps16*		LSC	887	817	890	877	876	885
*ycf3*	Intron1 Intron2	LSC	692	697	691	690	702	690
			744	739	739	739	739	739
*trnA-UGC*		IR	820	820	819	820	820	820
*trnG-U* *C* *C*		LSC	732	722	732	733	732	732
*trnI_GAU*		IR	780	781	780	775	780	781
*trnK-UUU*		LSC	2542	2502	2528	2538	2539	2535
*trnL-UAA*		LSC	438	423	438	438	438	438
*trnV-UAC*		LSC	573	573	573	573	573	573

## Supplementary Materials

The following are available online at https://www.mdpi.com/2223-7747/9/5/568/s1, Table S1. Predicted RNA-editing sites in the protein-coding genes of the mainland *Aster spathulifolius* chloroplast genome. Table S2. Analyses of the variable sites in the *Aster* chloroplast genomes. Table S3. Ka, Ks, and Ka/Ks values of 80 protein-coding genes from comparisons between mainland *A. spathulifolius* and five other *Aster* chloroplast genomes. Table S4. Number of simple sequence repeats and their positions in each *Aster* species. Table S5. Distribution of longer dispersed repeats in the six *Aster* chloroplast genome sequences. Table S6. Accession numbers of all the species used for the phylogenetic analysis and positive selected analysis. Table S7. Pairwise sequence divergence of the *Aster* chloroplast genomes. Table S8. The potential positive selection test based on the branch-site model. Figure S1. Comparison of the mainland *Aster spathulifolius* chloroplast genome with those of other *Aster* species using a CGView Comparison Tool analysis. From the outer ring: Ring 1: genes transcribed clockwise; Ring 2: genes transcribed counterclockwise; Ring 3: BLAST comparison of mainland *A. spathulifolius* and mainland *A. spathulifolius*; Ring 4: BLAST comparison of mainland *A. spathulifolius* and *A. tataricus*; Ring 5: BLAST comparison of mainland *A. spathulifolius* and *A. indicus*; Ring 6: BLAST comparison of mainland *A. spathulifolius* and *A. altaicus*; Ring 7: BLAST comparison of mainland *A. spathulifolius* and *A. hersileoides*; Ring 8: BLAST comparison of mainland *A. spathulifolius* and island *A. spathulifolius*; Ring 9: GC content; Ring 10: GCskew+; and Ring 11: GC skew–. Figure S2. Phylogenetic tree of nrITS sequences of various *Aster* species, generated using the maximum likelihood method. Numbers at nodes indicate bootstrap values (1000 repeats). The scale bar represents the expected number of nucleotide substitutions per site. Two *Diplostephium* species were used as the outgroup. The NCBI ID of sequences was shown next to species.
