# Supplementary figures and images for: Comparative Analysis of the Complete Chloroplast Genome of Mainland Aster spathulifolius and Other Aster Species
Source: Plants (Basel). 2020 Apr 29;9(5):568. doi: 10.3390/plants9050568 (PMC7285121; doi:10.3390/plants9050568)

Length: 152,732 bp

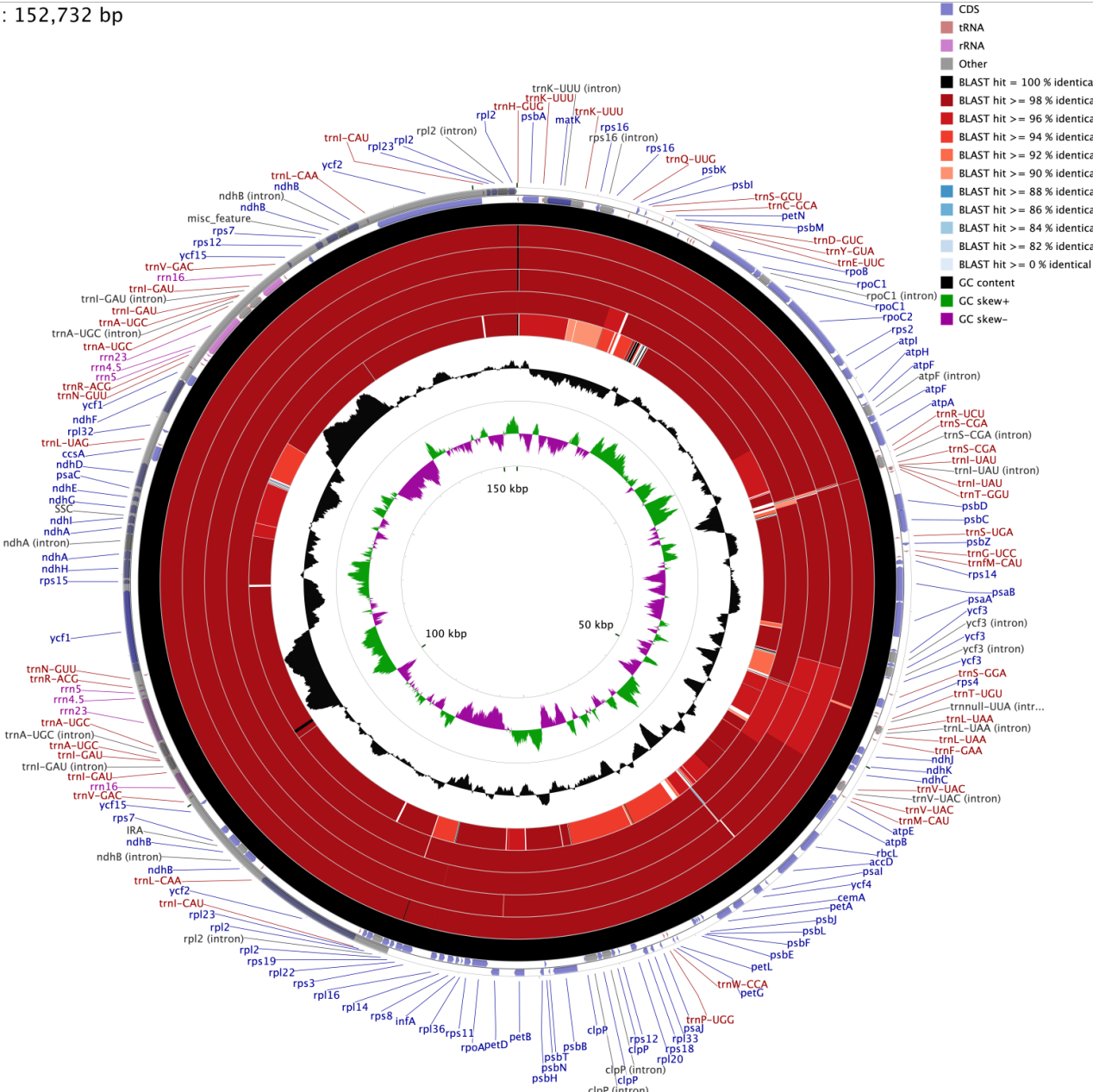

### Fig S1

Supplement: Supplementary file 1 [file plants-09-00568-s001.zip › Supplementary_figure.pdf]
